# Melatonin Regulates Glymphatic Function to Affect Cognitive Deficits, Behavioral Issues, and Blood–Brain Barrier Damage in Mice After Intracerebral Hemorrhage: Potential Links to Circadian Rhythms

**DOI:** 10.1111/cns.70289

**Published:** 2025-02-21

**Authors:** Yunzhao Chen, Hexi Guo, Xinguo Sun, Shanjun Wang, Mingyu Zhao, Junjie Gong, Anqi He, Jing Li, Yuheng Liu, Zengguang Wang

**Affiliations:** ^1^ Department of Neurosurgery, Tianjin Neurological Institute Tianjin Medical University General Hospital Tianjin China; ^2^ Department of Neurosurgery Inner Mongolia Autonomous Region People's Hospital Hohhot China; ^3^ Department of Neurosurgery Ordos Central Hospital Ordos China; ^4^ Department of Neurosurgery Binzhou People's Hospital Binzhou China; ^5^ Department of Neurosurgery Yidu Central Hospital of Weifang Qingzhou China

**Keywords:** blood–brain barrier, circadian rhythms, cognitive impairment, glymphatic system, intracerebral hemorrhage

## Abstract

**Background:**

Intracerebral hemorrhage (ICH) is a life‐threatening cerebrovascular disorder with no specific pharmacological treatment. ICH causes significant behavioral deficits and cognitive impairments. Recent research suggests that circadian rhythm regulation could be a promising therapeutic strategy for ICH. Melatonin has been shown to alleviate glymphatic system (GS) dysfunction by regulating circadian rhythms, thereby improving depressive‐like behaviors and postoperative sleep disorders in mice. However, its application in ICH treatment and specific mechanisms are not well understood.

**Methods:**

ICH models were created in 8‐to‐10‐week‐old mice using collagenase injection. Circadian rhythm modulation was tested with melatonin and luzindole. Behavioral and cognitive impairments were assessed with the modified neurological severity score, corner test, and novel object recognition test. Brain water content was measured by the dry/wet weight method, and cerebral perfusion was assessed by cerebral blood flow measurements. GS function was evaluated using RITC‐dextran and Evans blue assays. Immunofluorescence and western blotting were used to analyze GS function and BBB permeability.

**Results:**

Melatonin restored GS transport after ICH, promoting hematoma and edema absorption, reducing BBB damage, and improving cognitive and behavioral outcomes. However, luzindole partially blocked these benefits and reversed the neuroprotective effects.

**Conclusion:**

Melatonin and luzindole treatment affect GS function, BBB permeability, and cognitive‐behavioral outcomes in mice with ICH. The underlying mechanism may involve the regulation of circadian rhythms.

## Graphical abstract

Our study is the first to investigate the combined use of melatonin and luzindole for intracerebral hemorrhage‐induced adverse events. Melatonin alleviated glymphatic system dysfunction and blood–brain barrier damage, facilitated hematoma absorption and edema resolution, and mitigated cognitive‐behavioral deficits, while luzindole partially reversed these effects. This effect may be related to circadian rhythms.

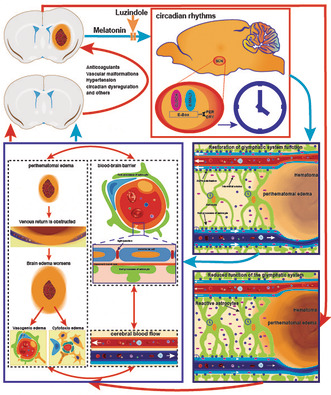

## 1 Introduction

Intracerebral hemorrhage (ICH) is a common and debilitating condition in neurosurgery [1, 2], and there are currently no specific therapeutic drugs [3]. The suddenly formed hematoma results in tearing of brain tissue and elevated intracranial pressure (ICP), which subsequently leads to perihematomal edema (PHE) [2, 4], reduced cerebral blood flow (CBF), blood–brain barrier (BBB) disruption [5, 6], and alterations in the intracranial microenvironment, triggering a cascade of amplifying responses. These effects may lead to severe behavioral deficits and cognitive dysfunction, profoundly impacting long‐term prognosis.

The glymphatic system (GS) facilitates the exchange of interstitial fluid (ISF) and cerebrospinal fluid (CSF) [7], aiding in the removal of intracranial metabolic waste, including inflammatory mediators, β‐amyloid (Aβ), Tau protein, and cellular debris, and maintaining central nervous system (CNS) homeostasis [8, 9]. Proper GS function relies on aquaporin‐4 (AQP4) on astrocytes [10], which is also critical for alleviating PHE [6]. Glymphatic blockade exacerbates the edema around the hematoma [11]. Disruption of GS function is associated with the development of neurological disorders such as Alzheimer's disease, traumatic brain injury, and ICH [11, 12, 13]. GS function exhibits circadian rhythms [14], with peak activity occurring during the sleep phases in mice [15]. AQP4 polarization is maximal during rest, and its loss affects circadian variations in GS influx and drainage to the deep cervical lymph nodes (dCLN) [15]. GFAP‐positive astrocytes are involved in both the generation of circadian rhythms and the pathogenesis of ICH [16, 17]. Circadian rhythms influence various critical processes, including sleep, brain metabolism, and the balance of chemical flux into and out of the brain [16]. Regulating circadian rhythms can not only improve sleep–wake disturbances, a risk factor for ICH [18], but also restore GS function, which may help clear the high levels of toxic substances produced by ICH, ultimately improving behavioral deficits and cognitive impairments resulting from ICH [19]. Thus, circadian rhythm regulation may represent a promising therapeutic target for ICH.

Melatonin, an endogenous circadian regulator, has been shown to reduce inflammation, oxidative stress, brain edema, and cognitive deficits [5, 20, 21], while restoring GS function [22]. Luzindole, a melatonin receptor antagonist, can work alongside melatonin to regulate circadian rhythms [23]. However, the combined effects of melatonin and luzindole on circadian rhythm regulation during ICH have not been elucidated. Disruption of circadian rhythms may exacerbate the severity and outcomes of ICH [24], while restoring circadian rhythms could facilitate recovery [17, 19, 25]. The specific mechanisms remain unclear. This study aims to investigate the effects of melatonin and luzindole on GS dysfunction, cerebral hematoma and edema, BBB damage, and cognitive‐behavioral deficits in a mouse model of ICH. Additionally, the study explores the potential of circadian rhythm regulation as a novel therapeutic strategy and target for ICH and other central nervous system disorders.

## 2 Materials and Methods

### 2.1 Animals

Male C57BL/6 mice (8–10 weeks, 22–24 g) from Beijing Vital River Laboratory were housed in a temperature‐controlled room with a 12‐h light/dark cycle and ad libitum access to food and water. Procedures were approved by Tianjin Medical University General Hospital's Animal Ethics Committee.

### 2.2 ICH Model

ICH model was prepared as described previously [26]. Using a microinjection pump (Shenzhen Ruiwode Life Science Technology Co. Ltd., China), 0.65 μL of Collagenase IV (Solarbio, C8160, 0.3 mg/mL) was injected at a rate of 0.5 μL/min. On the first day post‐surgery, mice without motor deficits were excluded from the study.

### 2.3 Experiment Design and Drug Administration

**FIGURE 1:**
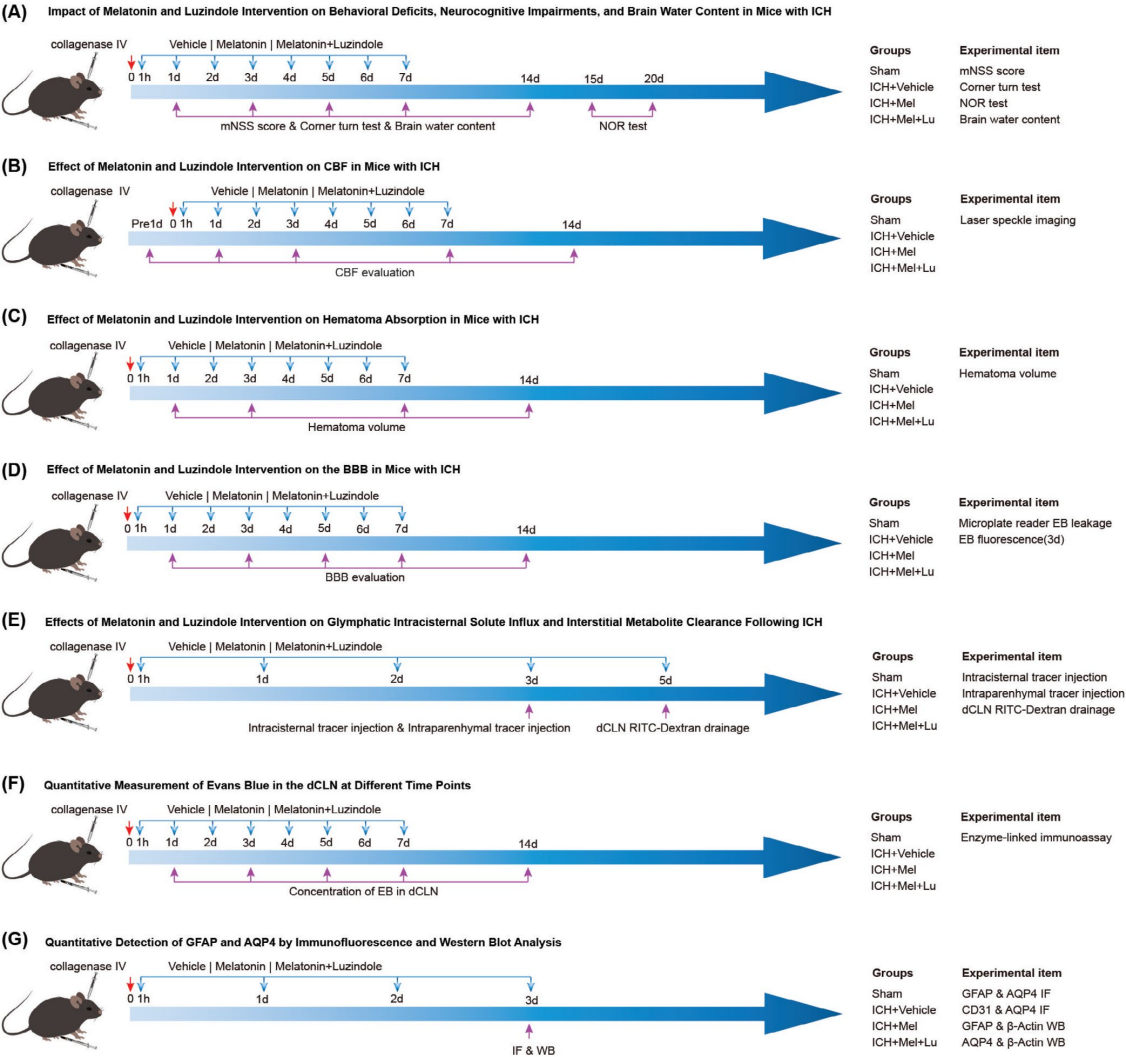
Experimental design and animal groups. AQP4, aquaporin 4; BBB, Blood–Brain Barrier; CBF, Cerebral Blood Flow; CD31, cluster of differentiation 31; DAPI, 4′,6‐diamidino‐2‐phenylindole; dCLN, deep cervical lymph nodes; EB, Evans blue; GFAP, glial fibrillary acidic protein; ICH, intracerebral hemorrhage; IF, immunofluorescence; Lu, luzindole; Mel, melatonin; mNSS, Modified neurological severity score; NOR test, novel object recognition test; RITC‐Dextran, Rhodamine B isothiocyanate dextran; WB, western blot.

Melatonin (L132758, Aladdin) was used to reset the circadian rhythm, with studies showing that a dose of 10 mg/kg is the most effective and safe with no side effects [21]. Luzindole (L132758, Aladdin, 30 mg/kg), a melatonin receptor antagonist, assessed circadian rhythms and responses. Mice were divided into four groups: Sham, ICH + Vehicle, ICH + Mel (ICH + Melatonin), and ICH + Mel+Lu (ICH + Melatonin+Luzindole) (Figure 1). Collagenase was injected to establish the ICH model except for the Sham group, which received saline. Melatonin and Luzindole were dissolved in DMSO and diluted with saline. ICH + Mel and ICH + Mel+Lu groups received daily intraperitoneal injections of melatonin or Luzindole, respectively. Sham and ICH groups received saline injections.

### 2.4 Behavioral Tests

Neurological function was assessed using the mNSS and corner test to evaluate motor, sensory, reflex, and balance functions [3]. The mNSS scores range from 0 (normal) to 18 (severe impairment). The corner test measures sensory, motor and postural asymmetry based on left turn percentages. Memory was evaluated with the novel object recognition (NOR) test [27, 28], where exploration times for familiar and novel objects were recorded and analyzed to compute the discrimination index. This can be expressed by the following equation: Discrimination index = (Tnovel − Tfamiliar)/(Tnovel + Tfamiliar).

### 2.5 Measurement of Brain Water Content

Brain water content was measured using a wet/dry method [3]. On days 1, 3, 5, 7, and 14 post‐ICH, brains were divided into the ipsilateral hemisphere, contralateral hemisphere, and cerebellum. The wet weight was measured, then the brains were dried at 100°C for 24 h. Brain water content = [(Wet Weight − Dry Weight)/Wet Weight] × 100%.

### 2.6 Laser Speckle Imaging

As previously described [29], CBF was assessed using the PeriCam PSI system (Perimed AB, Sweden) on the day before and on days 1, 3, 7, and 14 post‐ICH. Perfusion data were analyzed with PIMsoft 1.2 software, selecting five specific locations as regions of interest (ROI) for each sample. Average perfusion values were calculated.

### 2.7 Hematoma Volume Measurement

As described in previous research [3], mouse brains were collected on days 1, 3, 7, and 14 post‐ICH and sectioned into 1‐mm‐thick coronal slices. Hematoma volume was calculated using ImageJ software with the formula *V* = *t* × (S1 + S2 + … + Sn), where *V* represents the hematoma volume, *t* is the slice thickness, and S represents the hematoma area.

### 2.8 Assessment of BBB Permeability

To assess BBB permeability, two methods were employed: Evans blue (EB) extravasation absorbance measurement and fluorescence imaging [29, 30]. On days 1, 3, 5, 7, and 14 post‐modeling, 2% EB was injected via the tail vein and allowed to circulate for 2 h. Mice were then perfused with saline, and their brains were collected. The brain tissues were homogenized, centrifuged, and the absorbance of the supernatant was measured at 610 nm using a spectrophotometer. The concentration was quantified using a standard curve and normalized to tissue weight (μg/g).

For fluorescence imaging, 30‐μm thick coronal sections were prepared using a cryostat and stained with DAPI (blue). Fluorescence images of EB (red) were then captured using an inverted fluorescence microscope (1.25×/4×/10×). Finally, the permeability of the BBB was assessed by quantifying the percentage of the area occupied by red fluorescence (EB) relative to blue fluorescence (DAPI) using ImageJ software.

### 2.9 Intracisternal CSF Tracer Infusion

As previously described [28, 31], on day 3 post‐ICH, Hamilton 25 μL syringes and 33‐gauge needles were used to inject 10 μL of 2.5% 70 kDa rhodamine B isothiocyanate dextran (RITC‐dextran) at 1 μL/min into the cisterna magna, followed by a 30‐min circulation. Mice were then perfused, and brains were fixed overnight in 4% paraformaldehyde at 4°C. Coronal sections (100 μm thick) were prepared using a Leica Biosystems CM 1950 microtome. Fluorescence images were captured with an Olympus IX 73 inverted fluorescence microscope after DAPI staining. Quantitative analysis was conducted using ImageJ software.

### 2.10 Intraparenchymal Injections

To assess the clearance of interstitial metabolites in the brain [13], on day 3 post‐ICH, a Hamilton 5 μL syringe and a 33‐gauge needle were used to inject 0.5 μL of 2.5% 70 kDa RITC‐dextran at a rate of 0.2 μL/min into the hippocampus (2.0 mm posterior to bregma, 1.5 mm lateral to the right, 2 mm below the skull). One hour later, the mice were perfused with cold PBS, and the brains were extracted and fixed overnight in 4% paraformaldehyde at 4°C. Coronal sections (100 μm thick) were prepared, and fluorescent images were captured after DAPI staining. Quantitative analysis was performed using ImageJ software.

### 2.11 EB Colorimetric Method for dCLN

As previously described [26], on days 1, 3, 5, 7, and 14 post‐ICH, mice were anesthetized, and 8 μL of 2% EB was injected into the cerebellomedullary cistern at a rate of 2 μL/min. Cervical tissues were then dissected to expose dCLN, which were subsequently collected. Representative images of EB drainage from the dCLN were captured using an Olympus SZX‐7 microscope. We collected all dCLNs from both the left and right sides of each mouse. If a mouse had more than one dCLN on either side, all dCLNs from that side were pooled together and treated as a single data point for analysis. The dCLNs were homogenized, centrifuged, and the supernatant was transferred to a 96‐well plate. EB concentration was measured at 610 nm, and actual EB concentrations were calculated from a standard curve using Microsoft Excel.

### 2.12 Ex Vivo Imaging of Fluorescent CSF‐Tracers

To further investigate the drainage of dCLN on day 5 post‐ICH, bilateral dCLN were extracted 30 min after the injection of RITC‐dextran. The dCLN were stained with DAPI, and representative fluorescence images were captured using an Olympus IX 73 microscope [32]. The dCLN were then sectioned into 10 μm thick frozen slices, restained with DAPI, and imaged again. Fluorescence areas of RITC‐dextran (red) and DAPI (blue) were quantified using ImageJ software, and the percentage of the red area relative to the blue area was calculated using Microsoft Excel.

### 2.13 Immunofluorescence

As previously described [31], on day 3 post‐ICH, brain sections were analyzed by immunofluorescence. Following extraction, brain tissue was dehydrated in a gradient series of 15% and 30% sucrose solutions. The tissue was then embedded in OCT compound (Sakura Finetek USA) and sectioned into 10 μm coronal slices. Sections were blocked for 1.5 h with PBS containing 3% bovine serum albumin, 0.2% Triton X‐100, and 0.05% Tween 20, and then incubated overnight at 4°C with primary antibodies: rabbit anti‐AQP4 (1:800, 59678, Cell Signaling), mouse mAb GFAP (1:500, 3670S, Cell Signaling), and goat anti‐CD31 (1:500, AF3628, Bio‐Techne). Subsequently, slices were incubated for 1 h with species‐specific fluorescently labeled secondary antibodies. After DAPI staining, fluorescence images were captured and analyzed using ImageJ software.

### 2.14 AQP4 Polarization Evaluation

AQP4 polarization was assessed on day 3 post‐ICH [12, 33]. ImageJ was used to quantify AQP4 polarization levels in blood vessels and surrounding regions, with ROI chosen manually in a blinded manner. The fluorescence intensity of AQP4 labeling was measured along a 100 μm‐long axis perpendicular to the direction of the blood vessel, generating a linear fluorescence profile extending from the brain tissue into the vessel and back into the surrounding brain tissue. Threshold analysis was used to measure the percentage area of AQP4 immunofluorescence greater than or equal to that surrounding the blood vessel (AQP4% area). Polarization was represented as the percentage area where AQP4 immunoreactivity was lower than that surrounding the vessel (1 − AQP4% area).

### 2.15 Western Blot

As previously described [28], protein samples from the brain tissue and hippocampus were collected on day 3 post‐ICH. The proteins were transferred to polyvinylidene fluoride (PVDF, Millipore) membranes, which were then blocked with 5% non‐fat milk. The membranes were incubated overnight at 4°C with the following primary antibodies: AQP4 (1:1000, sc‐32739, Santa Cruz), GFAP (1:1000, Rabbit mAb #12389S, Cell Signaling), and β‐Actin (1:2000, TA‐09, ZSGB‐BIO). Following this, the membranes were incubated with secondary antibodies for 1 h. Protein bands were visualized using the ChemiDoc Touch Imaging System and quantified using ImageJ software.

### 2.16 Statistical Analysis

All data were statistically analyzed using GraphPad Prism 10.3. Data are presented as mean ± standard error of the mean (SEM). The normality of data distribution was evaluated using the Shapiro–Wilk test. If the data were normally distributed, One‐way analysis of variance (ANOVA) was used to assess group differences. For data that were not normally distributed, non‐parametric tests (Kruskal–Wallis test) were applied. Two‐way ANOVA and Tukey's post hoc test were employed to evaluate the differences among multiple groups with repeated measurements. A *p*‐value less than 0.05 was considered statistically significant.

## 3 Results

### 3.1 Melatonin Treatment Alleviated Cognitive and Behavioral Impairments, Reduced Brain Edema, and Mitigated the Decrease in CBF, While Luzindole Partially Blocked These Therapeutic Effects

**FIGURE 2:**
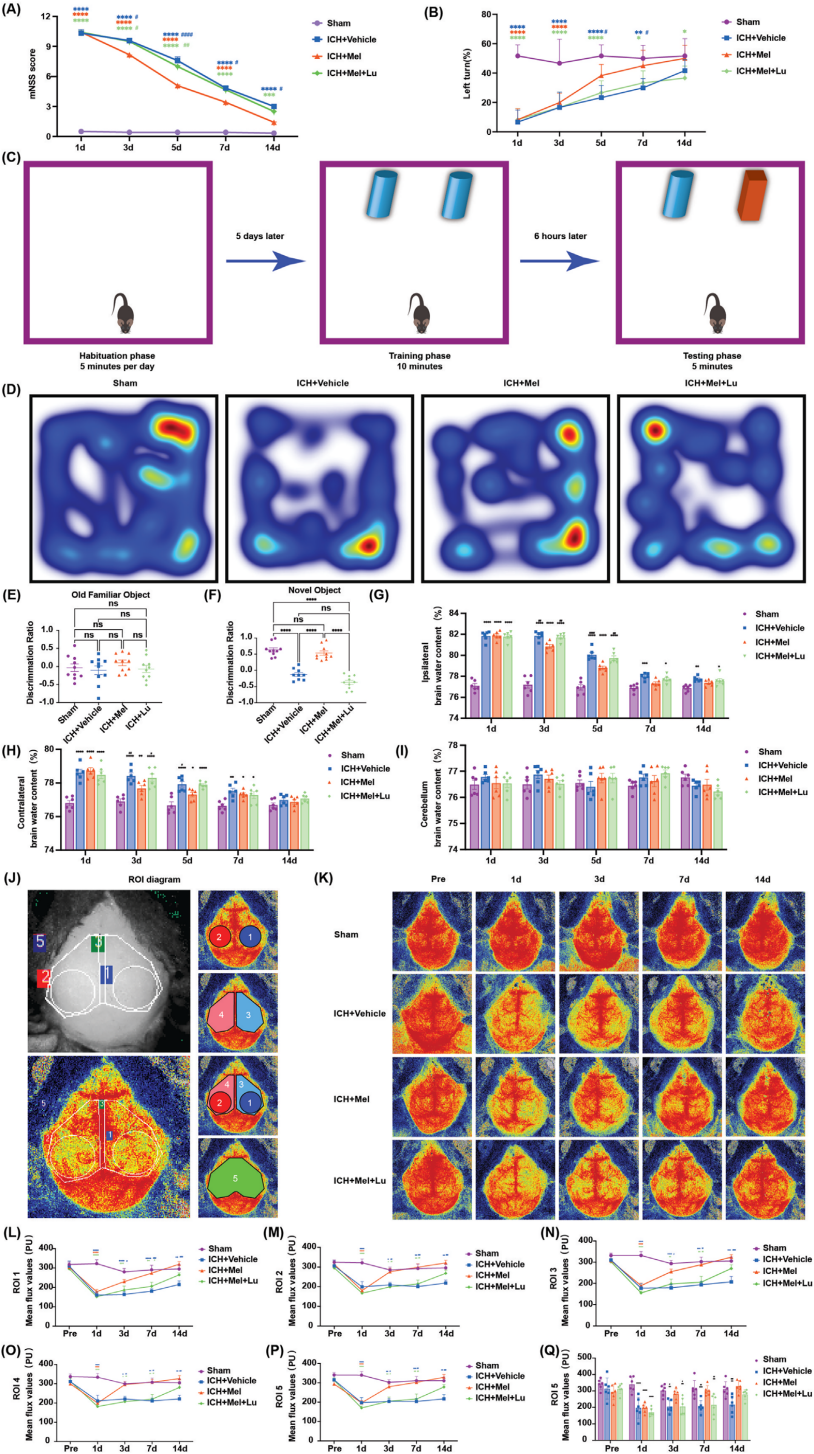
Melatonin treatment alleviated cognitive and behavioral impairments and brain edema, and mitigated the reduction in CBF, while luzindole partially blocked the therapeutic effects. (A) Motor function is assessed by mNSS score (*n* = 6 per group). (B) Motor function is assessed by the corner turn test (*n* = 6 per group). (C) Schematic representation of the method and process for the NOR test. (D) Representative heatmap of explored objects of the NOR test. (E, F) NOR test discrimination ratio of mice (In the study, *n* = 10 for all groups, except for the ICH + Vehicle group, which had *n* = 9 due to one mouse dying before the test). (G–I) Brain water content of ipsilateral hemisphere (G), contralateral hemisphere (H) and the cerebellum (I) (*n* = 6 per group). (J) Laser speckle imaging schematic of the ROI. (K) Representative images of CBF by LSCI in different groups at different time points. (L–O) Quantitative analysis of continuous CBF changes before and after ICH in the ipsilateral ROI 1 (L) and ROI 3 (N), and the contralateral ROI 2 (M) and ROI 4 (O) (*n* = 6 per group). (P, Q) Quantitative analysis of continuous CBF changes in whole cerebral cortex ROI 5 before and after ICH (*n* = 6 per group). All data are shown as mean ± SEM. *p < 0.05 versus sham group, **p < 0.01 versus sham group, ***p < 0.001 versus sham group, ****p < 0.0001 versus sham group, ^#^ *p* < 0.05 versus ICH + Mel group, ^##^ *p* < 0.01 versus ICH + Mel group, ^###^ *p* < 0.001 versus ICH + Mel group, ^####^ *p* < 0.0001 versus ICH + Mel group. CBF, Cerebral Blood Flow; ICH, intracerebral hemorrhage; LSCI, Laser Speckle Contrast Imaging; Lu, luzindole; Mel, melatonin; mNSS, Modified neurological severity score; NOR test, novel object recognition test; ROI, regions of interest.

Early behavioral deficits and cognitive impairments are common following ICH and are associated with poor outcomes [34, 35]. To assess the effects of biological regulation on motor function in ICH mice, we performed the mNSS (Figure 2A) and the Corner Test (Figure 2B). Significant behavioral deficits were observed in all experimental groups within 24 h post‐ICH. The mNSS showed that melatonin treatment accelerated recovery compared to the ICH + Vehicle group, with differences evident from days 3 to 14, and the most pronounced on day 5. Luzindole inhibited the therapeutic effects of melatonin. Although trends were similar, the mNSS appeared to be more sensitive than the Corner Test in assessing behavioral deficits.

To evaluate the impact of melatonin and luzindole treatment on cognitive impairments in ICH mice, we conducted the NOR test (Figure 2C–F). Results indicated that the ICH + Vehicle group spent significantly less time around the novel object compared to the ICH + Mel group (Figure 2F). Melatonin treatment significantly increased the time spent exploring the novel object. However, pre‐treatment with luzindole negated this improvement (Figure 2F). Overall, these data suggest that melatonin has neuroprotective effects on cognitive impairments induced by ICH, but luzindole can reverse these effects.

Brain edema plays a key role in the pathophysiology following ICH and is closely associated with disturbances in GS function [36]. To explore how melatonin regulates brain edema through its effects on the GS, we specifically measured brain water content (Figure 2G–I). The results showed that changes in brain water content were consistent with behavioral assessments, with significant differences observed between the melatonin and vehicle groups from days 3 to 7, peaking on day 5. Luzindole treatment reversed these effects. The most pronounced changes were seen in the ipsilateral brain (Figure 2G), followed by the contralateral brain (Figure 2H), while no significant differences were noted in the cerebellum (Figure 2I). These findings indicate a strong association between brain edema and behavioral impairments.

Additionally, since the maintenance of GS and BBB functions is closely related to CBF [6], we employed laser speckle imaging to evaluate CBF changes before and after ICH (Figure 2J–Q). Baseline CBF measurements were taken before ICH. CBF significantly decreased on day 1 post‐ICH and began to recover by day 3. The ICH + Mel group exhibited faster recovery compared to the ICH + Vehicle group, with significant differences noted on days 3, 7, and 14. Luzindole treatment moderately inhibited this recovery (Figure 2L–Q). By day 14, CBF in the ICH + Mel group was higher than in the sham group, possibly due to vascular proliferation, while the sham group also experienced mild damage from vehicle injection. Overall, melatonin treatment improved cortical CBF following ICH, whereas luzindole diminished this therapeutic effect.

### 3.2 Melatonin Treatment Enhances Hematoma Absorption and Reduces BBB Permeability, While Luzindole Partially Blocked This Effect

**FIGURE 3:**
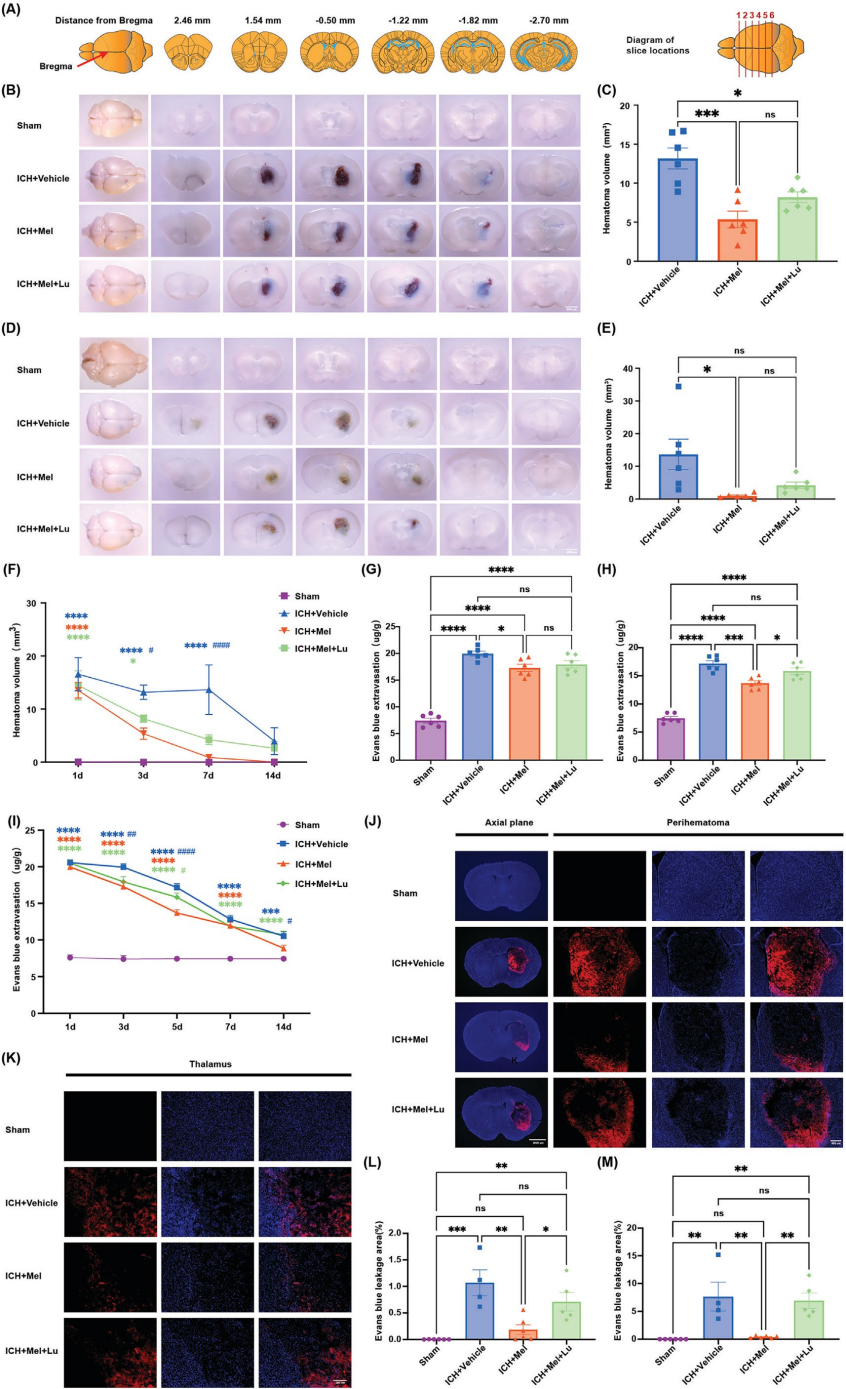
Melatonin treatment enhances hematoma absorption and reduces BBB permeability, while luzindole partially blocked this effect. (A) Schematic of mouse brain sections. (B) Representative horizontal and coronal images of brains at 3 days after ICH. Scale bars: 2000 μm (C) Quantitative analysis of hematoma volume on day 3 post‐ICH (*n* = 6 per group). (D) Representative horizontal and coronal images of brains on day 7 post‐ICH. Scale bars: 200 μm. (E) Quantitative analysis of hematoma volume on day 7 post‐ICH (*n* = 6 per group). (F) Trends in hematoma absorption area at 1, 3, 7, and 14 days in the four groups (*n* = 6 per group). (G, H) Quantitative analysis of EB leakage intensity at 3 days (G) and 5 days (H) after ICH (*n* = 6 per group). (I) Four groups' trends in EB leakage intensity at 1, 3, 5, 7, and 14 days (*n* = 6 per group). (J) Fluorescence microscopy images of the hematoma area were examined on the third day after ICH to assess BBB integrity using the EB permeability assay. Axial plane, Scale bars: 2000 μm. Perihematoma, Scale bars: 400 μm. (K) Fluorescence microscopy images of the thalamus were examined on the third day after ICH to assess BBB integrity using the EB permeability assay. Scale bars: 200 μm. (L, M) Quantification of EB fluorescence intensity in the hematoma area (L) and thalamus (M) (*n* = 4–6 per group). All data are shown as mean ± SEM. *p < 0.05 versus sham group, **p < 0.01 versus sham group, ***p < 0.001 versus sham group, ****p < 0.0001 versus sham group, ^#^ *p* < 0.05 versus ICH + Mel group, ^##^ *p* < 0.01 versus ICH + Mel group, ^###^ *p* < 0.001 versus ICH + Mel group, ^####^ *p* < 0.0001 versus ICH + Mel group. BBB, blood–brain barrier; EB, Evans blue;ICH, intracerebral hemorrhage; Lu, luzindole; Mel, melatonin.

We assessed hematoma absorption in ICH mice by measuring hematoma volume in brain slices at days 1, 3, 7, and 14 post‐ICH (Figure 3A–F). BBB damage and repair were evaluated by EB extravasation using a spectrophotometer (Figure 3G–I) and EB fluorescence staining (Figure 3J–M). Mice in the ICH + Mel group showed smaller hematoma volumes on days 3 and 7 compared to the ICH + Vehicle group, indicating that melatonin significantly enhanced hematoma absorption. No significant difference was observed between the ICH + Mel and the ICH + Mel + Lu groups, suggesting luzindole did not strongly inhibit melatonin's effects. EB extravasation results showed that melatonin treatment reduced EB leakage on days 3 and 5 compared to the ICH + Vehicle group (Figure 3G,H). On day 5, luzindole significantly inhibited melatonin's positive effects (Figure 3H), though not on day 3 (Figure 3G). This suggests that melatonin may significantly promote BBB repair in the early stages, while the inhibitory effect of luzindole may require time to accumulate and become more pronounced in the later stages, thereby suppressing this repair process. EB fluorescence staining on day 3 showed significant differences between the ICH + Mel and the ICH + Mel + Lu groups (Figure 3L,M), indicating its sensitivity compared to spectrophotometric measurements, making it easier to detect earlier changes.

### 3.3 Melatonin and Luzindole Treatment Affects Glymphatic Intracisternal Solute Influx After ICH

**FIGURE 4:**
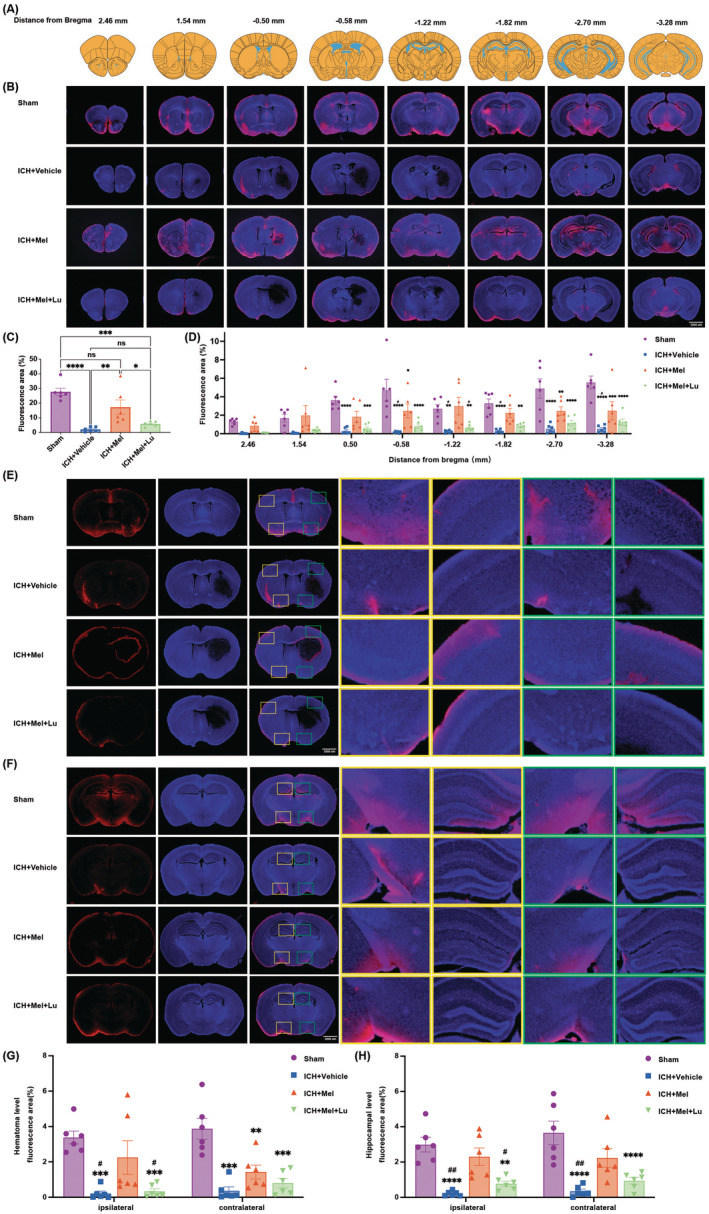
Melatonin and luzindole treatment affects glymphatic intracisternal solute influx after ICH. (A) Schematic of mouse brain sections. (B) Representative brain sections stained for nuclei (DAPI; blue) and RITC‐dextran (red) influx into eight coronal brain sections of mice from four groups. Scale bar: 2000 μm. (C) Quantification of the percentage of RITC‐dextran covered area fraction in brain sections in (B) (*n* = 6 per group). (D) Quantitative analysis and inter‐group comparison of fluorescence tracer coverage area in brain slices from different distances to the bregma across the four groups (*n* = 6 per group). (E) Representative images of fluorescent tracer inflow function at the left or right side of the maximal hematoma plane in the four groups. Scale bar: 2000 μm. (F) Representative images of fluorescent tracer inflow function at the left or right side of the hippocampal plane in the four groups. Scale bar: 2000 μm. (G, H) Quantitative analysis was performed to compare the differences between the four groups in the percentage of RITC‐dextran covered area in ipsilateral or contralateral brain sections at the hematoma maximum (G) and hippocampal (H) levels (*n* = 6 per group). All data are shown as mean ± SEM. *p < 0.05 versus sham group, **p < 0.01 versus sham group, ***p < 0.001 versus sham group, ****p < 0.0001 versus sham group, ^#^ *p* < 0.05 versus ICH + Mel group, ^##^ *p* < 0.01 versus ICH + Mel group. DAPI, 4′,6‐diamidino‐2‐phenylindole; ICH, intracerebral hemorrhage; Lu, luzindole; Mel, melatonin; RITC‐Dextran, Rhodamine B isothiocyanate dextran.

To investigate the changes in CSF influx following ICH and the effects of melatonin and luzindole treatment on this function, RITC‐dextran was injected into the cisterna magna on day 3 post‐ICH (Figure 4A,B). The functionality of GS was assessed by observing tracer accumulation. Quantitative analysis (Figure 4C) revealed a significant reduction in CSF tracer penetration into the brain. In the ICH + Mel group, CSF tracer influx was significantly increased, whereas a significant statistical difference was also observed between the ICH + Mel + Lu and ICH + Vehicle groups, indicating that melatonin treatment improved GS influx function, while luzindole treatment reversed this effect.

We also compared the influx function across different brain sections (Figure 4D), finding significant regional differences in recovery. In the hematoma sections (Figure 4E) and hippocampal sections (Figure 4F), the ICH + Mel group exhibited significantly more fluorescence in the ipsilateral hematoma sections compared to the ICH + Vehicle group, while luzindole inhibited this therapeutic effect. Melatonin treatment significantly affected both hemispheres in the hippocampal sections (Figure 4H), but in the hematoma sections, it had a significant effect only on the ipsilateral side with no significant difference on the contralateral side (Figure 4G). This indicates that melatonin treatment effectively promotes recovery of influx function in the ipsilateral hematoma region and significantly improves influx function in both hemispheres of the hippocampus.

### 3.4 Melatonin and Luzindole Treatment Affects the Interstitial Metabolite Clearance Pathway After ICH

**FIGURE 5:**
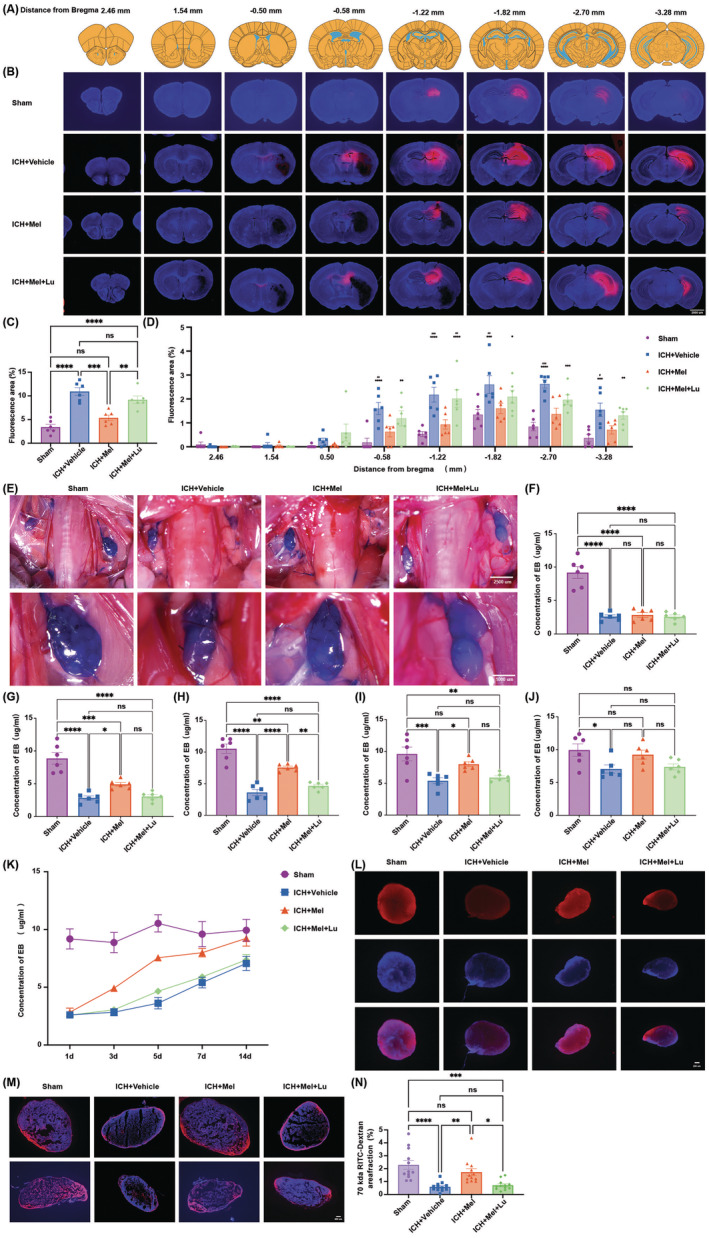
Melatonin and luzindole treatment affects the interstitial metabolite clearance pathway after ICH. (A) Schematic of mouse brain sections. (B) Representative brain sections stained for nuclei (DAPI; blue) and RITC‐dextran (red) clearance from the brain parenchyma of mice from four groups. Scale bar: 2000 μm. (C) Quantification of the percentage of RITC‐dextran covered area fraction in brain sections in (B) (*n* = 6 per group). (D) Quantitative analysis and inter‐group comparison of fluorescence tracer coverage area in brain slices from different distances to the bregma across the four groups (*n* = 6 per group). (E) Representative images of the distribution of EB in the dCLN of each group on day 5 after treatment. Upper panel, Scale bar: 2500 μm. Lower panel, Scale bar: 1000 μm. (F–J) Quantification of EB concentration in the dCLN was performed using a microplate reader on days 1 (F), 3 (G), 5 (H), 7 (I), and 14 (J) post‐treatment (*n* = 6 per group). (K) Trends in EB concentration in dCLN on days 1, 3, 5, 7, and 14 post‐treatment (*n* = 6 per group). (L) Representative images of whole dCLN showing drainage of RITC‐Dextran. Scale bar: 200 μm. (M) Representative images of dCLN section showing drainage of RITC‐Dextran. Scale bar: 200 μm. (N) Graph depicting percent area of RITC‐Dextran coverage of dCLN (*n* = 6 per group). All data are shown as mean ± SEM. *p < 0.05 versus sham group, **p < 0.01 versus sham group, ***p < 0.001 versus sham group, ****p < 0.0001 versus sham group, ^#^ *p* < 0.05 versus ICH + Mel group, ^##^ *p* < 0.01 versus ICH + Mel group, ^###^ *p* < 0.001 versus ICH + Mel group. DAPI, 4′,6‐diamidino‐2‐phenylindole; dCLN, deep cervical lymph nodes; EB, Evans blue; ICH, intracerebral hemorrhage; Lu, luzindole; Mel, melatonin; RITC‐Dextran, Rhodamine B isothiocyanate dextran.

To investigate the impact of melatonin and luzindole treatment on CSF influx following ICH, we injected RITC‐dextran into the hippocampus and assessed interstitial solute clearance by comparing residual tracer levels across different experimental groups (Figure 5A,B). Quantitative analysis (Figure 5C) revealed that the ICH + Vehicle group showed a significantly increased tracer retention in brain tissues, whereas the ICH + Mel group exhibited a substantial reduction in tracer retention. Luzindole partially reversed the beneficial effects of melatonin. Significant differences in solute clearance recovery were observed across various brain sections (Figure 5D), indicating variable contributions of brain regions to solute clearance.

Recent studies highlight the dCLN as key sites for brain solute clearance. To quantitatively assess CSF solute clearance, we performed EB absorbance assays on dCLN (Figure 5E). Results showed a significant decrease in EB excretion starting from day 1 post‐ICH (Figure 5F). By days 3, 5, and 7, the ICH + Mel group exhibited faster EB excretion recovery compared to the ICH group, with the most pronounced difference on day 5 (Figure 5G–I). Luzindole significantly inhibited this effect (Figure 5H). By day 14 post‐ICH, EB excretion in the untreated ICH + Vehicle group remained significantly different from the sham group, while no significant differences were observed between the sham group and either the ICH + Mel or ICH + Mel + Lu groups (Figure 5J). Melatonin treatment showed a trend of faster recovery (Figure 5K). To further validate these results, we collected dCLN from mice 30 min after RITC‐dextran injection into the cisterna magna on day 5 post‐ICH and performed immunofluorescence detection (Figure 5L,M). Results confirmed that the ICH + Mel group had higher fluorescence values compared to the ICH + Vehicle group, while luzindole treatment significantly suppressed this effect (Figure 5N). In summary, melatonin treatment significantly accelerated solute elimination, whereas luzindole partially counteracted this effect.

### 3.5 Melatonin Treatment Reduced the Expression of Reactive GFAP and Improved the Polarization of AQP4, While Luzindole Partially Blocked This Effect

**FIGURE 6:**
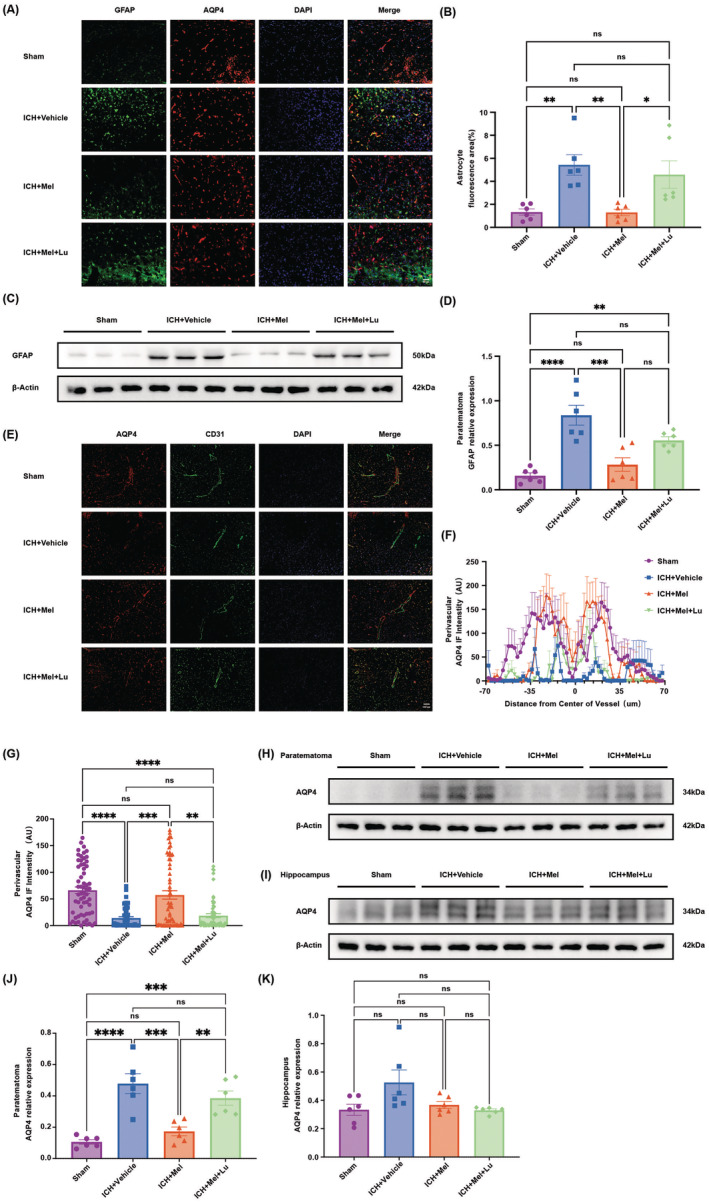
Melatonin treatment reduced the expression of reactive GFAP and improved AQP4 polarization, while luzindole partially inhibited the effectiveness of this treatment. (A) Coimmunofluorescence staining for AQP4 (red) and GFAP (green) around the lesion. Scale bar: 50 μm. (B) Quantification of the percentage of GFAP covered area fraction in brain sections (*n* = 6 mice per group). (C) Representative western blot images of GFAP and β‐actin expression 3 days after ICH (*n* = 6 mice per group). (D) Quantification of relative protein expression of GFAP normalized to β‐actin optical density. (E) Coimmunofluorescence staining for AQP4 (red) and CD31 (green) around the lesion. Scale bar: 100 μm. (F) AQP4 immunofluorescence projections across large cortical vessels were quantified. (G) Quantification of AQP4 expression surrounding large cortical vessels (0–1 μm from the vessel wall). (H) Representative western blot scans of AQP4 protein expression in tissue adjacent to the hematoma. (I) Quantification of relative protein expression of AQP4 in tissue adjacent to the hematoma, normalized to β‐actin optical density. (J) Representative western blot scans of AQP4 protein expression in the hippocampus. (K) Quantification of relative protein expression of AQP4 in the hippocampus, normalized to β‐actin optical density. All data are shown as mean ± SEM. *p < 0.05, **p < 0.01, ***p < 0.001, ****p < 0.0001. AQP4, aquaporin 4; CD31, cluster of differentiation 31; DAPI, 4′,6‐diamidino‐2‐phenylindole; GFAP, glial fibrillary acidic protein; ICH, intracerebral hemorrhage; IF, immunofluorescence; Lu, luzindole; Mel, melatonin; WB, western blot.

Evidence indicates that the function of GS depends on astrocytic end‐feet AQP4, and co‐staining of AQP4 and GFAP is commonly used to assess reactive astrocyte proliferation. Our results (Figure 6A,B) show that on day 3 post‐ICH, GFAP expression is significantly higher in the ICH + Vehicle group compared to the sham group. Melatonin treatment significantly reduces GFAP levels, while luzindole partially reverses this effect. Similar results were observed in GFAP Western blot experiments (Figures 6C,D and S1A–D), although no significant difference was found between the ICH + Mel and ICH + Mel + Lu groups. AQP4 polarization analysis (Figure 6E–G) indicates that melatonin treatment restores AQP4 polarization around blood vessels, whereas luzindole partially inhibits this trend. Western blot analysis (Figures 6H,J and S2A–D) shows that melatonin significantly restores AQP4 accumulation in peri‐hematoma tissues, while luzindole inhibits this therapeutic effect. No significant differences were observed in AQP4 Western blot results among the groups in the hippocampus (Figures 6I,K and S3A–D).

## 4 Discussion

The circadian clock is a complex regulatory system that controls the daily physiological rhythms of all organisms, known as the circadian rhythm, through rhythm proteins (such as Clock, Bmal1, Per1, Per2, Cry1, and Cry2) in a transcriptional feedback loop [16, 37]. In mammals, the suprachiasmatic nucleus (SCN) in the hypothalamus functions as the central pacemaker, and melatonin, synthesized in the pineal gland, regulates the circadian rhythm by binding to melatonin receptors MT1 and MT2 [38, 39]. While evidence connects circadian dysfunction with brain disorders, causal relationships remain unclear [40]. Advancements in circadian biology may enable targeted disease prevention and treatment [41], as circadian rhythms influence brain waste clearance [16]. Previous studies have demonstrated that melatonin, by regulating circadian rhythms, can restore GS function and the normal rhythmic expression of rhythm proteins in a chronic unpredictable mild stress mouse model, thereby improving depressive‐like behaviors [42]. Based on this background, our study investigates the effects of melatonin and luzindole on outcomes following ICH, focusing on their impact on hematoma absorption, brain edema resolution, BBB permeability, and GS function.

Approximately 75% of patients with ICH suffer from motor, sensory, language, and other higher neurological deficits [1, 4]. Secondary brain injury (SBI) leads to damage of BBB integrity, brain edema, and reduced CBF [4]. Timely resolution of hematoma can mitigate SBI [1]. ICH commonly occurs in the basal ganglia (striatum), where lesions can cause significant learning and memory deficits. Melatonin treatment has been shown to reduce long‐term brain atrophy and improve cognitive impairments in mice by modulating glymphatic function and sleep structure [22, 42]. Our study demonstrates that melatonin treatment significantly ameliorates behavioral deficits and cognitive impairments in ICH mice, although luzindole partially inhibits its therapeutic effects.

PHE leads to elevated ICP, reduces CBF and cerebral perfusion, and increases BBB permeability, which further impedes edema resolution and results in SBI [2, 43, 44]. Melatonin, known for its anti‐edema properties [45], alleviates severe brain edema and behavioral disorders induced by ICH [4]. Our findings demonstrate that melatonin treatment significantly reduced brain edema and facilitated the recovery of CBF.

The permeability of BBB, formed by endothelial cells interconnected by tight junction proteins, is regulated by circadian rhythms and sleep [15, 46, 47, 48]. Disruption of the BBB following ICH is associated with poor prognosis [49]. Studies indicate that melatonin can alleviate BBB disruption after ICH and indirectly facilitate hematoma resolution [4, 16], consistent with our results. Our study demonstrates that melatonin promotes hematoma absorption and BBB repair, with the restoration of circadian rhythms potentially serving as the underlying mechanism through which melatonin exerts its therapeutic effects.

The GS, mediated by AQP4 on astrocytic end‐feet, facilitates the exchange of CSF and brain tissue fluid, rapidly removing brain metabolites and maintaining brain homeostasis [8, 10, 50]. Enhancing GS function can improve hematoma resolution after ICH [26, 51], which supports our findings. We demonstrated that melatonin treatment significantly enhances glymphatic influx in the brain, while luzindole may interfere with melatonin's regulatory effects.

GS transports CSF and solutes into the brain via the peri‐arterial route, while ISF and solutes are removed from the brain through the peri‐venous route [52]. The dCLN are a key exit point for removing harmful metabolic waste via GS [53]. The influx of CSF and solute clearance to dCLN within GS is regulated by endogenous circadian rhythms [15, 54]. Sleep deprivation reduces glymphatic clearance, leading to the accumulation of metabolic waste in the brain [53]. Melatonin enhances waste clearance during non‐rapid eye movement (NREM) sleep and enhances the clearance of amyloid‐beta (Aβ) to the dCLN [24, 53]. Our study demonstrates that melatonin significantly reduces residual fluorescence in brain tissue and increases EB concentration in the dCLN, indicating an enhanced capability for solute transport to these dCLN.

Astrocyte function involves metabolic coupling with neurons and neurotransmitter clearance [16]. Inhibiting astrocyte proliferation represents a novel target for improving glymphatic pathways [55, 56]. Normal AQP4 polarization is crucial for GS's regulation of brain fluid balance and waste clearance [57]. Enhancing AQP4 polarization can promote glymphatic transport [58]. Melatonin maintains the circadian rhythm of astrocytic AQP4 polarization by regulating the expression of the circadian rhythm protein Per2 [42]. Our study found that melatonin treatment facilitated the restoration of AQP4 polarization and significantly reduced excessive GFAP expression, indicating that melatonin improves glymphatic function. This finding corroborates results from other models involving melatonin [4, 45]. Given that previous research has demonstrated that melatonin restores the normal rhythmic expression of circadian proteins, we hypothesize that the restoration of normal circadian rhythms following ICH may be a potential mechanism through which melatonin exerts its beneficial therapeutic effects.

Our study is the first to investigate the combined application of melatonin and luzindole in the treatment of adverse events induced by ICH. We demonstrated that melatonin improved the GS's CSF influx and solute clearance to some extent, facilitated hematoma absorption and edema resolution, and alleviated BBB disruption and cognitive‐behavioral deficits. The observed effects of melatonin and luzindole may be related to their potential role in regulating circadian rhythms. However, our study has certain limitations. First, the molecular mechanisms underlying the combined regulation of circadian rhythms by melatonin and luzindole remain to be further explored. This will be a focus of our future research. Second, we only evaluated the effects of short‐term treatment with melatonin and luzindole, while treatment following ICH should be a long‐term process. Finally, although clinical studies have demonstrated the role of circadian rhythms in stroke treatment, neuroprotective strategies that have been effective in rodent stroke models have not yet yielded positive results in clinical trials, likely due to differences in circadian rhythms between rodents and humans [59, 60]. Therefore, translating the findings from animal model studies on the effects of circadian rhythms on neuroprotection into clinical applications may require further exploratory research.

## 5 Conclusions

Melatonin may exert neuroprotective effects in ICH by alleviating GS dysfunction and BBB damage, while luzindole can reverse these effects. Regulation of the circadian rhythm may underlie this mechanism. Therefore, circadian rhythm modulation holds promise as a potential therapeutic strategy for treating ICH.

## Author Contributions

Yunzhao Chen, Zengguang Wang, and Hexi Guo conceptualized and designed the study. Yunzhao Chen drafted the manuscript, with revisions made by Zengguang Wang and Yuheng Liu, and all authors approved the final version. Yunzhao Chen, Hexi Guo, and Xinguo Sun performed model preparation and CSF tracer infusion. Yunzhao Chen, Shanjun Wang, Mingyu Zhao, and Junjie Gong conducted immunofluorescence, Western blot experiments, and EB leakage assays. Xinguo Sun, Shanjun Wang, Anqi He, and Jing Li carried out brain water content measurements and behavioral testing. Yuheng Liu performed statistical analysis of the data.

## Conflicts of Interest

The authors declare no conflicts of interest.

## Supporting information


Data S1.

## Data Availability

The data that support the findings of this study are available from the corresponding author upon reasonable request.
