# Supplementary material for: Melatonin Regulates Glymphatic Function to Affect Cognitive Deficits, Behavioral Issues, and Blood–Brain Barrier Damage in Mice After Intracerebral Hemorrhage: Potential Links to Circadian Rhythms
Source: CNS Neurosci Ther. 2025 Feb 21;31(2):e70289. doi: 10.1111/cns.70289 (PMC11843476; doi:10.1111/cns.70289)
Supplement: Supplementary file 1 — Data S1. [file CNS-31-e70289-s001.pdf]

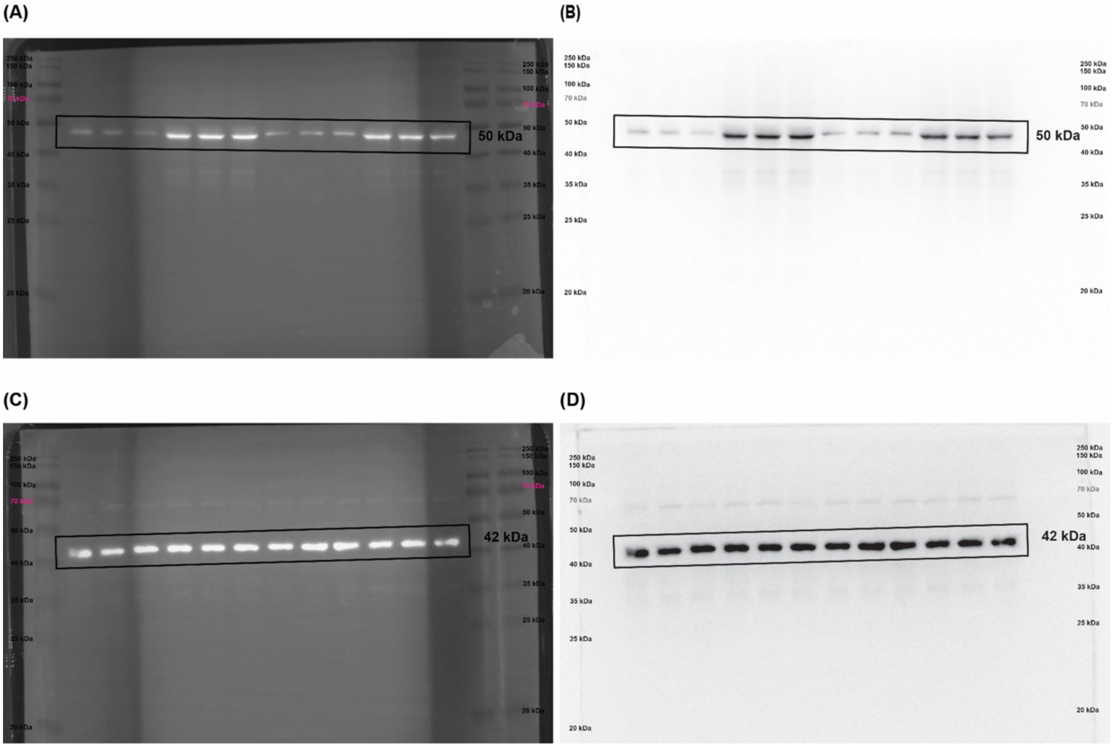

FIGURE S1 Full unedited gel/blot for Figure 6C. (A) and (B) Representative western blot images of GFAP expression on day 3 post-ICH. (C) and (D) Representative western blot images of β-Actin expression on day 3 post-ICH.

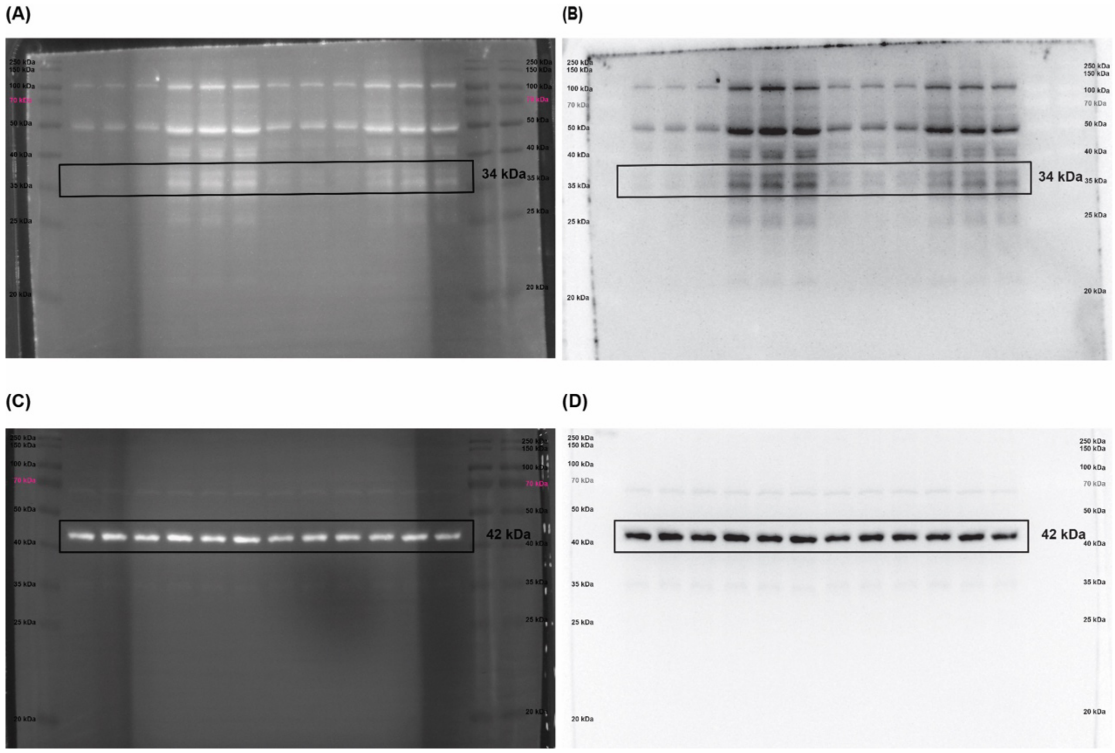

FIGURE S2 Full unedited gel/blot for Figure 6H. (A) and (B) Representative western blot images of AQP4 expression on day 3 post-ICH. (C) and (D) Representative western blot images of β-Actin expression on day 3 post-ICH.

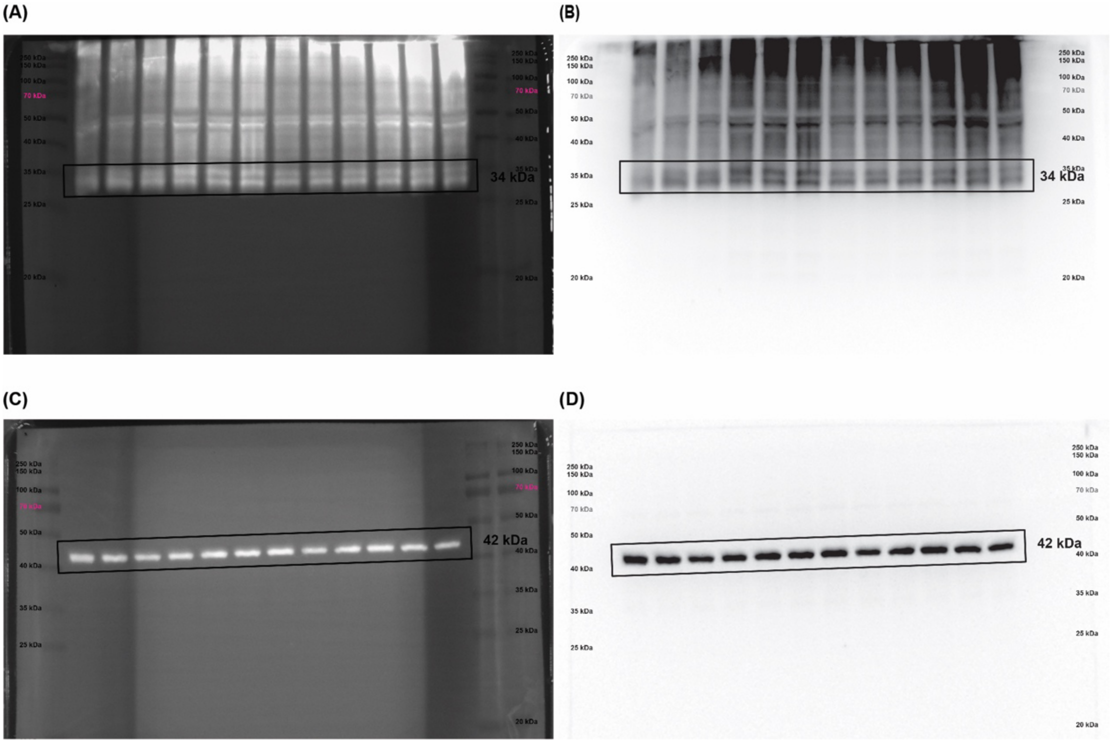

FIGURE S3 Full unedited gel/blot for Figure 6I. (A) and (B) Representative western blot images of AQP4 expression on day 3 post-ICH. (C) and (D) Representative western blot images of β-Actin expression on day 3 post-ICH.
